# Predicting preterm birth using machine learning techniques in oral microbiome

**DOI:** 10.1038/s41598-023-48466-x

**Published:** 2023-11-30

**Authors:** You Mi Hong, Jaewoong Lee, Dong Hyu Cho, Jung Hun Jeon, Jihoon Kang, Min-Gul Kim, Semin Lee, Jin Kyu Kim

**Affiliations:** 1 Department of Obstetrics and Gynecology, University of Ulsan College of Medicine, Asan Medical Center, Seoul, Republic of Korea; 2 Department of Biomedical Engineering, Ulsan National Institute of Science and Technology (UNIST), Ulsan, Republic of Korea; 3 Department of Obstetrics and Gynecology, Jeonbuk National University Medical School, Jeonju, Republic of Korea; 4 Research Institute of Clinical Medicine of Jeonbuk National University–Biomedical, Research Institute of Jeonbuk National University Hospital, Jeonju, Republic of Korea; 5 Helixco Inc., 50, Unist-gil, Eonyang-eup, Ulju-gun, Ulsan, Republic of Korea; 6 Department of Pharmacology, Jeonbuk National University Medical School, 20, Geonji-ro, Deokjin-gu, Jeonju, Jeollabuk-do Republic of Korea; 7 Department of Pediatrics, Jeonbuk National University Medical School, 20, Geonji-ro, Deokjin-gu, Jeonju-si, Jeollabuk-do Republic of Korea

**Keywords:** Computational biology and bioinformatics, Diseases

## Abstract

Preterm birth prediction is essential for improving neonatal outcomes. While many machine learning techniques have been applied to predict preterm birth using health records, inflammatory markers, and vaginal microbiome data, the role of prenatal oral microbiome remains unclear. This study aimed to compare oral microbiome compositions between a preterm and a full-term birth group, identify oral microbiome associated with preterm birth, and develop a preterm birth prediction model using machine learning of oral microbiome compositions. Participants included singleton pregnant women admitted to Jeonbuk National University Hospital between 2019 and 2021. Subjects were divided into a preterm and a full-term birth group based on pregnancy outcomes. Oral microbiome samples were collected using mouthwash within 24 h before delivery and 16S ribosomal RNA sequencing was performed to analyze taxonomy. Differentially abundant taxa were identified using DESeq2. A random forest classifier was applied to predict preterm birth based on the oral microbiome. A total of 59 women participated in this study, with 30 in the preterm birth group and 29 in the full-term birth group. There was no significant difference in maternal clinical characteristics between the preterm and the full-birth group. Twenty-five differentially abundant taxa were identified, including 22 full-term birth-enriched taxa and 3 preterm birth-enriched taxa. The random forest classifier achieved high balanced accuracies (0.765 ± 0.071) using the 9 most important taxa. Our study identified 25 differentially abundant taxa that could differentiate preterm and full-term birth groups. A preterm birth prediction model was developed using machine learning of oral microbiome compositions in mouthwash samples. Findings of this study suggest the potential of using oral microbiome for predicting preterm birth. Further multi-center and larger studies are required to validate our results before clinical applications.

## Introduction

Preterm birth (PTB), defined as delivery of newborns before 37 weeks of gestation, is a leading cause of morbidity and mortality in newborns^1^. Established risk factors for PTB include genitourinary tract infections, short cervical length, and multiple pregnancies^2^. However, there is still disagreement regarding the magnitude of these factors’ effects on birth outcomes. Early identification of pregnant women at high risk of PTB can facilitate the implementation of strategies to prolong gestation and improve birth outcomes^3^.

Despite increased understanding of risk factors contributing to PTB, there remains a considerable lack of sensitivity in predictive models that can serve as a framework for intervention strategies^4^. Numerous attempts have been made to predict PTB using machine learning techniques combined with data from health records, inflammatory markers, and vaginal microbiome^5^. Fetal fibronectin is widely used clinically due to its affordability and simplicity. However, it has a low prediction rate with a sensitivity of only 56%^6^. Cervical length measurement also has limitations due to the hassle and inaccuracy of the procedure and the need for a skilled specialist^7^.

Approximately 70% of PTBs result from spontaneous onset of preterm labor and preterm pre-labor rupture of membranes due to intrauterine infection and inflammation^8^. However, the mechanism of PTB cannot be fully explained by inflammatory and infectious pathways as anti-inflammatory and antibiotic treatments could not reduce PTB rates^9^. With advancements in molecular genetic technology, studies on maternal microbiomes using 16S ribosomal RNA (rRNA) sequencing have emerged to explore unknown pathways of PTB^10^.

Microorganisms associated with PTB have been postulated to originate from one of two places: the reproductive or genitourinary tract ascending through the cervix or a hematogenous route^11^. Recent research has identified vaginal microbial signatures in women who later experience PTB and attempted to predict PTB using cervicovaginal fluid^12^. Although existing reports have verified a potential relationship of vaginal microbiome with PTB, they can only explain an ascending route.

Decades of epidemiological research studies have suggested that periodontitis is an independent risk factor for various adverse birth outcomes, including PTB^13^. Based on these precedents, it is expected that the oral microbiome can explain another hematogenous route. However, prenatal oral microbiome is not well understood^14^.

Thus, this study aimed to compare oral microbiome compositions between a PTB group and a full-term birth group, to identify oral microbiome associated with PTB, and to develop a machine learning prediction model of PTB based on oral microbiome compositions.

## Methods

### Study design and participants

This study was conducted on singleton pregnant women admitted for delivery at Jeonbuk National University Hospital between 2019 and 2021. This study received approval from the Ethical Research Committee (IRB file No. 2019-01-024). All participants provided written informed consent. Eligible participants included women admitted for induction delivery, elective cesarean section, and those who were hospitalized due to symptoms of preterm labor or preterm pre-labor rupture of membranes.

### Data collection and grouping

Data on current and historical pregnancy outcomes were collected from questionnaires and electronic medical records. This information encompassed demographic factors (gestational age, birth weight, sex) and maternal risk factors (maternal age at delivery, cesarean section, preterm pre-labor rupture of membranes, previous preterm delivery history, gestational or overt diabetes mellitus, pregnancy-induced or chronic hypertension, and pre-pregnancy overweight or gestational weight gain). All subjects were divided into a PTB group or a full-term birth (FTB) group, with PTB defined as delivery before 37 weeks of gestation.

### Oral microbiome sample collection

Oral microbiome samples were collected using mouthwash within 24 h before delivery. Standard sterile techniques were employed. Medical staff supervised all sample collection procedures. Participants were instructed to avoid brushing their teeth, eating, or drinking 30 min before sampling. Saliva samples were obtained by rinsing the mouth with 12 mL of a gargle solution (E-zen Gargle, JN Pharm, Pyeongtaek, Korea) for 30 s. Sample were labeled with the subject’s anonymous ID and stored at 4 °C until further processing. The resuspended sample was transferred to a microcentrifuge tube. Genomic DNA was extracted using an Exgene™ Clinic SV kit (GeneAll Biotechnology, Seoul, Korea) according to the manufacturer’s instructions and stored at − 20 °C.

### 16S rRNA gene sequencing and taxonomy assignment

Specimens were sent to Department of Biomedical Engineering, Ulsan National Institute of Science and Technology for taxonomy assignment. Then 16S rRNA sequencing was performed using an Illumina MiSeq Reagent Kit v3 (Illumina, San Diego, CA, USA) commissioned by Macrogen (Macrogen, Seoul, Korea). Library protocols for amplifying V3 and V4 regions were used. Pooled library was sequenced using a v3 600 cycle chemistry after diluting the sample to a final concentration of 6 pM with 20% PhiX control to generate 300 bp paired end reads.

### Bioinformatics and statistical analysis

Independent t-test and chi-square test were used to compare differences between the PTB group and the FTB group. Statistical Package for Social Sciences version 20.0 was used for all data analyses. Statistical significance was considered at $p<0.05$.

16S rRNA sequences from study subjects were imported with Quantitative Insights Into Microbial Ecology (version 2022.2) for further processing^14^. Sequences were filtered with DADA2^15^. Amplicon sequence variants were assigned taxonomy with the Human Oral Microbiome Database (version 15.22)^16^.

To measure richness of microbiomes, two diversity indices were calculated. Alpha diversity, a measure of species within a particular community, was calculated using the Faith’s phylogenetic diversity index within the Quantitative Insights Into Microbial Ecology platform. Communities numerically dominated by a few species will exhibit a low Faith’s phylogenetic diversity index, whereas communities in which abundance is distributed equally among species will exhibit a high evenness. Mann–Whitney–Wilcoxon test was used to find statistically significant differences in Faith’s phylogenetic diversity index. Beta diversity measures dissimilarity between pairs of communities. It was calculated using the Hamming diversity index. PERMANOVA multivariate test was used to calculate statistically significant differences in the Hamming diversity index.

To identify differentially abundant taxa (DAT) with distinct abundance differences between the PTB group and the FTB group, DESeq2 was implemented using “DESeqDataSetFromMatrix” method as described in the package tutorial^17^. Taxa with $\left|{\mathrm{log}}_{2}{\text{FoldChange}}\right|>1$ and $\text{adjusted }{\text{p}}<0.05$ were considered as significantly different.

### Machine learning prediction model development

Following qualitative and quantitative analyses of associations between the PTB group and each bacterium, a random forest classifier was used to find the criteria to predict PTB with oral microbiome data. Random forest classification is an ensemble machine learning algorithm that summarizes many decision trees to improve classification evaluations and robustness^18^. Random forest classifier was implemented to predict PTB based on oral microbiome compositions. To assure consistence and reliable classification results, we performed stratified $k$-fold cross-validation ($k=5$). Moreover, to decide the best features that could maximize classification evaluations, we performed random forest classification evaluations only with some DAT selected by their importances. Evaluations for classification included accuracy, balanced accuracy, precision, sensitivity, and specificity.

### Ethics approval and consent to participate

The research protocol was approved by the Institutional Review Board of Jeonbuk National University Hospital (No. 2019-01-024) and was performed according to the Declaration of Helsinki.

## Results

### Study participant demographics

In this study, a total of 69 volunteer mothers were initially recruited. However, one participant with incomplete data and nine individuals with twin pregnancies were excluded from the study cohort. As a result, 59 women (30 in the PTB group and 29 in the FTB group) were included in the final analysis. Demographic and clinical characteristics of subjects in the PTB group and the FTB group are summarized in Table 1. Because prelabor rupture of membrane (PROM) is a major cause of preterm birth, it was significantly higher in PTB group. There was no significant difference in other maternal clinical characteristics between the PTB group and the FTB group. There were no cases with a history of smoking or concurrent periodontal disease in both groups.

**Table 1:** Baseline clinical characteristics of study subjects.

	PTB (n = 30)	FTB (n = 29)	P
Maternal age	31.8 ± 5.2	33.7 ± 4.5	0.687
Cesarean section, n (%)	20 (66.7)	24 (82.7)	0.233
Previous preterm birth history, n (%)	4 (13.3)	1 (3.4)	0.353
Prelabor rupture of membrane, n (%)	12 (40)	1 (3.4)	0.001
Pre-pregnant overweight, n (%)	8 (26.7)	7 (24.1)	1.000
Gestational weight gain, g	9.0 ± 5.9	11.5 ± 4.6	0.262
Gestational or overt diabetes, n (%)	2 (6.7)	2 (6.9)	1.000
Pregnancy-induced or chronic hypertension, n (%)	11 (36.7)	4 (13.8)	0.072
Gestational age, wks	32.5 ± 3.4	38.3 ± 1.1	< 0.001
Birth weight, g	1973.4 ± 686.6	3283.4 ± 402.7	< 0.001
Male, n (%)	14 (46.7)	13 (44.8)	1.000

Mean ± standard deviation.

continuous variable for Independent t-test.

categorical variable for Pearson’s Chi-square test.

### Comparison of oral microbiomes

The oral microbiome comprised 13,953,804 sequences from 59 oral microbiome samples, with 102,305.95 ± 19,095.60 and 64,823.41 ± 15,841.65 reads per sample before and after filtering low-quality reads and trimming extra-long tails, respectively. After filtering low-quality reads and trimming extra-long tails, remaining representative reads were clustered in amplicon sequence variants with their exact sequence match. There were no significant differences in measures of alpha diversity (Faith’s phylogenetic diversity index) and beta diversity (Hamming distance) indices for samples between the PTB group and the FTB group (Supplementary Fig. 1).

**Figure 1:**
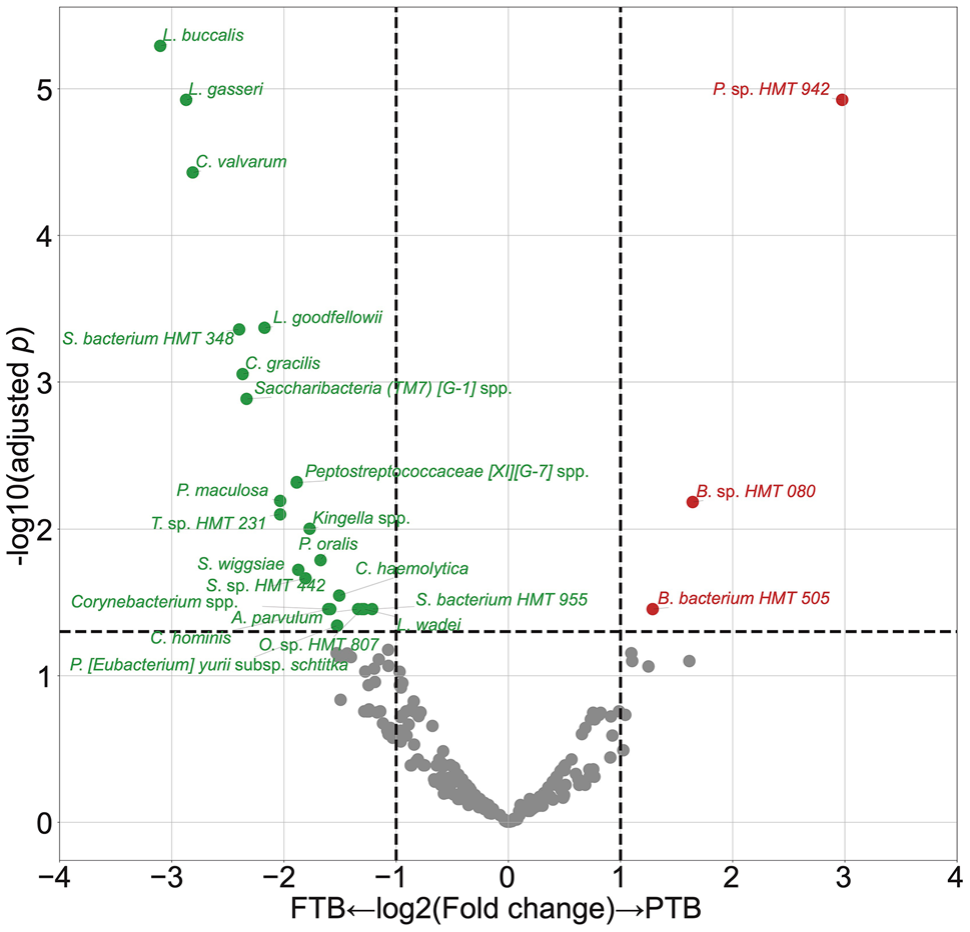
DAT volcano plots. Volcano plot shows DAT, with PTB-enriched DAT shown as red dots and FTB-enriched DAT shown as green dots. Taxa with $\left|{\mathrm{log}}_{2}{\text{FoldChange}}\right|>1$ and $\text{adjusted p}<0.05$ are considered as significantly different. DAT, Differentially abundant taxa; PTB, Preterm birth; FTB: Full-term birth.

Of 465 genera and species analyzed, 32 DAT between the PTB group and the FTB group were selected by DESeq2, including 26 FTB-enriched DAT and six PTB-enriched DAT. In order to mitigate the confounding effect of PROM, we excluded 7 PROM-related DATs from these 32 DAT (Supplementary Fig. 2). There were a total of 25 DATs between the PTB group and the FTB group, with 22 DATs enriched in the FTB group and 3 in the PTB group, as depicted in Fig. 1.

**Figure 2:**
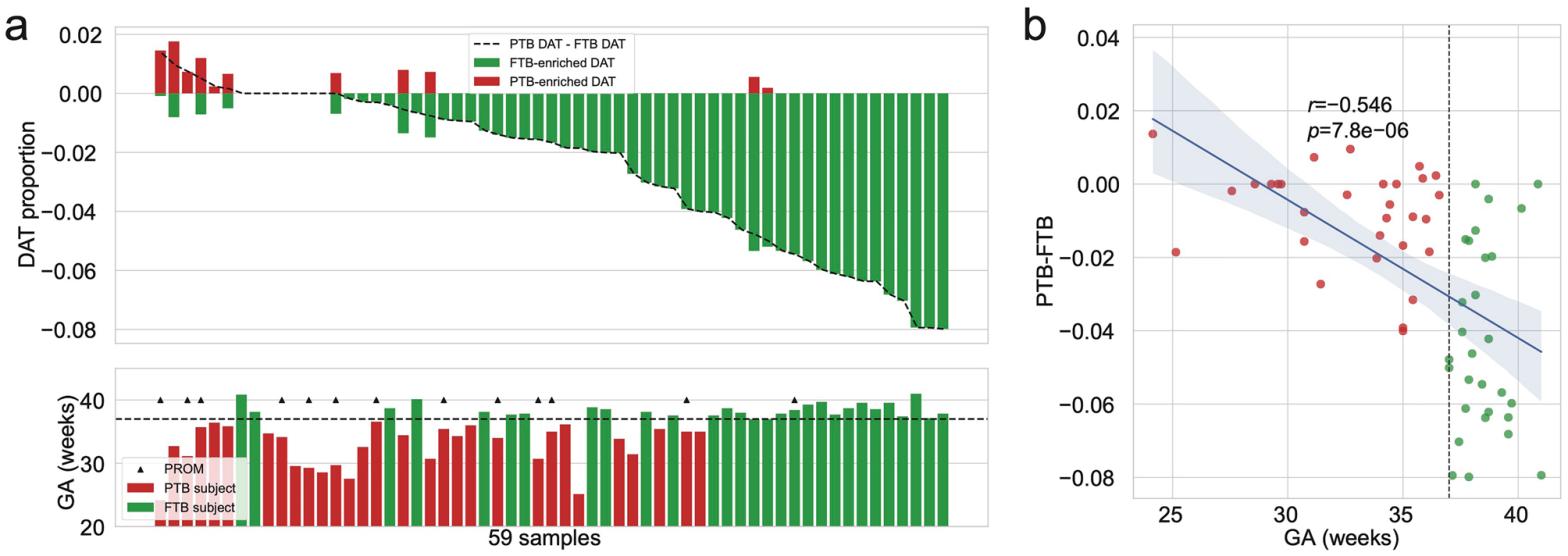
Oral microbiome compositions over DAT. (**a**) Proportions of DAT of study subjects. Samples are sorted by difference between PTB-enriched DAT proportion and FTB-enriched DAT proportion. GA of samples are shown, matched with order of upper panel. PTB: red bar, FTB: green bar. PROM: arrow head. (**b**) Correlation plot with GA and difference between PTB-enriched DAT proportion and FTB-enriched DAT proportion. Pearson correlation shows strong negative coefficient (r = − 0.542) between GA and difference between PTB-enriched DAT proportion and FTB-enriched DAT proportion. DAT, Differentially abundant taxa; PTB, Preterm birth; FTB: Full-term birth; PROM: prelabor rupture of membrane; GA, Gestational age.

Figure 2 displays DAT volcano plot in the oral microbiome sorted by differences between FTB-enriched DAT and PTB-enriched DAT proportions, indicating a decrease in gestational age for FTB-enriched DAT proportions. Pearson correlation analysis revealed a strong negative correlation ($r=-0.542$) between gestational age and difference between PTB-enriched DAT and FTB-enriched DAT proportions.

### Random forest classification

**Figure 3:**
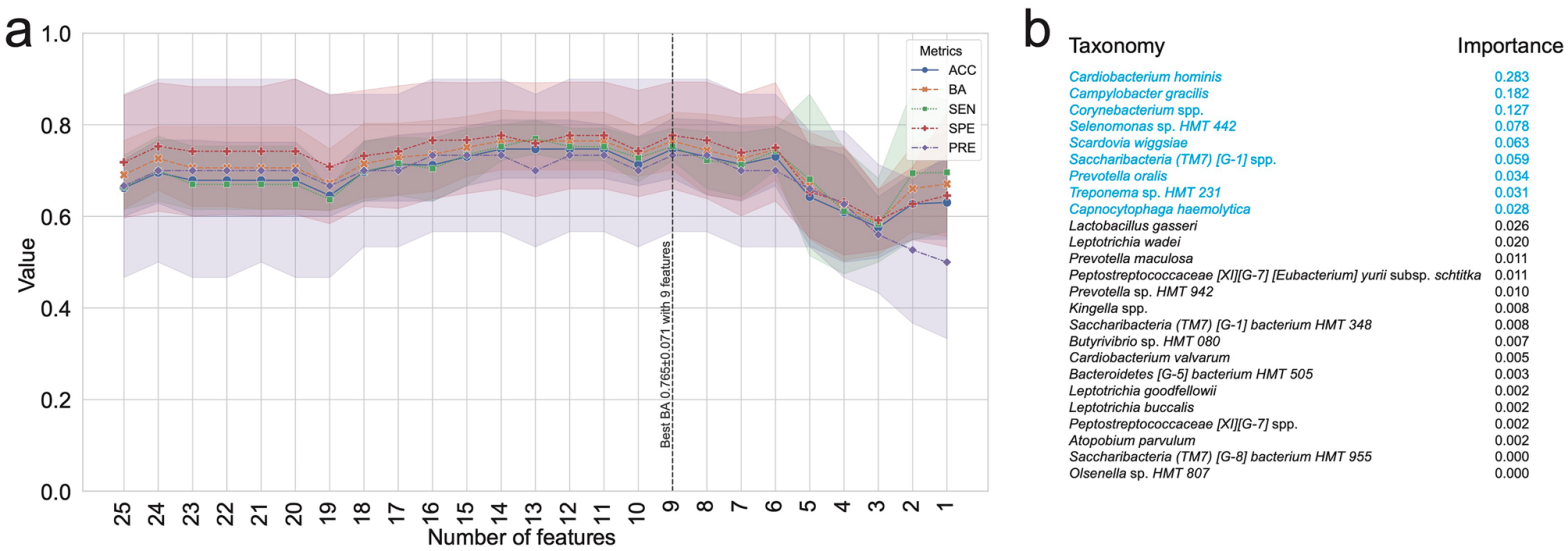
Machine learning evaluations over DAT. (**a**) Machine learning evaluation upon number of features (DAT). Random Forest classifier has the best balanced accuracy (0.765 ± 0.071) with the 9 most important DAT. (**b**) Importance of DAT is shown. Note that 0 ≤  importance  ≤ 1 and $\sum {\text{importance}}=1$. The 20 most important DAT that give the best balanced accuracy is marked with blue. DAT, Differentially abundant taxa; ACC, Accuracy; BA, balanced accuracy; SEN, Sensitivity; SPE, Specificity; PRE, precision.

Random forest classifiers were established to classify PTB based on DAT. The best balanced accuracy (0.765 ± 0.071) was achieved using 9 most important taxa (Fig. 3a). The Random forest model calculated the importance of each DAT (Fig. 3b). Overall accuracy, precision, sensitivity, and specificity were 0.714 ± 0.061, 0.700 ± 0.194, 0.728 ± 0.058, and 0.743 ± 0.138, respectively. In order to validate the performance of our machine learning prediction model, we conducted a validation test on 9 twin pregnancies that were excluded from the paper (Supplementary Fig. 3). On PTB subjects of these twin samples, the machine learning classifications have 87.5% accuracy, comparable to the machine learning classification on the singleton study subjects.

## Discussion

In this study, we developed a method for predicting PTB based on random forest classifier using oral microbiome compositions. Recently, several sporadic reports have suggested a bidirectional relationship between oral microbiome and pregnancy^11^. However, prenatal oral microbiome is not well understood yet. Some research has shown that oral microbial dysbiosis combined with gingival inflammation can lead to adverse pregnancy outcomes, including low birth weight, PTB, preeclampsia, and miscarriages^19^. Nevertheless, these results have been inconsistent due to methodologies employed in studies that only target known pathogens.

*Fusobacterium nucleatum* is the most prevalent oral microbiome studied. *Fusobacterium nucleatum* is a Gram-negative, anaerobic, filamentous oral microbiome. It is considered one of the most abundant species in the oral microbiome. It can also be isolated from vaginal microbiome^20,21^. Intra-amniotic *Fusobacterium nucleatum* infection leading to PTB has been reported in human and animal studies^22^. Other studies have shown that other oral pathogens including *Porphyromonas gingivalis* and intrauterine *Bergeyella* spp. can be isolated from the placenta of women who deliver prematurely^23,24^. In the present study, although *Bergeyella* spp. was overrepresented in the PTB group with PROM, it was excluded in the finally selected 25 DATs. Furthermore, *Campylobacter gracilis* was one of the FTB-enriched DAT that can aid colonization by periodontal pathogens including *Porpyhromonas gingivalis* in subgingival microbiome^25^. *Lactobacillus gasseri* was also one of the FTB-enriched DAT. It is known that *Lactobacillus gasseri* in vaginal microbiome can decrease early PTB risk^21,26,27^.

We found that decisive species differentiating between two groups were mainly abundant in the FTB group, with DAT consisting of 22 FTB-enriched DAT and 3 PTB-enriched DAT. We hypothesize that deficiency of species having a protective impact might have triggered the pathophysiology of PTB. Two different mechanisms have been proposed to explain the relationship between unhealthy microbiota composition and adverse pregnancy outcomes. The first mechanism proposed that periodontal bacteria originating in the gingival biofilm could translocate from the unhealthy oral cavity and cross the placenta, reach the intra-amniotic fluid and fetal circulation and directly affect the fetoplacental unit, resulting in bacteremia^28^. The second mechanisms proposed that systemic dissemination of endotoxins and/or inflammatory mediators derived from periodontal plaque and secreted by the subgingival inflammatory site could be carried to the fetoplacental unit^29,30^. Although certain microbiota has the same species, their subgroups can have both positive and negative influences on pregnancy outcomes. Following this line of thought, we believe that composition or dysbiosis of the oral microbiome is more important than the presence of specific microbiota.

It is worth nothing that microbial changes occurring during pregnancy might be nature consequences of a healthy pregnancy. Three reasons can explain the susceptibility to oral diseases such as periodontitis during pregnancy. These diseases are common in pregnant women due to hormonally driven hyper-reactivity of the gingiva to bacteria in the subgingival biofilm. Other factors that increase the risk of poor oral health during pregnancy include changes in dietary habits (frequent snacking or increased consumption of carbohydrate-rich foods), stomach acids from nausea and vomiting that contribute to the breakdown of tooth enamel, and a decreased likelihood of seeking dental care during pregnancy. We plan to implement pathway analyses to investigate direct link between the microbiome and PTB.

Even though there was limited power resulting from a small number of participants and restricted validation sample size, our study verified that oral microbiota might provide potential biomarkers for predicting pregnancy complications using machine learning methods including random forest classification. Additionally, the fact that the entire microbiome was not analyzed was a limitation of this study because our analysis only used relative values measured by 16S rRNA sequencing, not 16S metagenome sequencing. We did not investigate other factors could impact the oral microbiome, such as participant’s diet and socioeconomic status.

Despite these limitations, this prospective study demonstrated the potential of a PTB prediction model using oral microbiome in mouthwash. Further multi-center and larger-scale studies are needed to confirm out results before applying techniques developed in this study in the clinical field.

### Supplementary Information

Supplementary Figure 1.

Supplementary Figure 2.

Supplementary Figure 3.

## Data Availability

Sequences for all 59 samples are deposited in the Sequence Read Archives under the project ID PRJNA985119 (https://dataview.ncbi.nlm.nih.gov/object/PRJNA985119?reviewer=6fdj2e9c8gp9vtf52n330e2h8j). All codes used during this study are available on GitHub at https://github.com/CompbioLabUnist/Helixco_Premature. The Docker image used throughout this study is available in DockerHub at https://hub.docker.com/r/fumire/helixco_premature.

## Abbreviations

PTB: Preterm birth
rRNA: Ribosomal RNA
FTB: Full-term birth
DAT: Differentially abundant taxa
PROM: Prelabor rupture of membrane

## References

[1] Blencowe H, Cousens S, Oestergaard MZ, Chou D, Moller AB, Narwal R (2012). National, regional, and worldwide estimates of preterm birth rates in the year 2010 with time trends since 1990 for selected countries: A systematic analysis and implications. Lancet.

[2] Goldenberg RL, Culhane JF, Iams JD, Romero R (2008). Epidemiology and causes of preterm birth. Lancet.

[3] Iams JD, Berghella V (2010). Care for women with prior preterm birth. Am. J. Obstet. Gynecol..

[4] Sotiriadis A, Papatheodorou S, Kavvadias A, Makrydimas G (2010). Transvaginal cervical length measurement for prediction of preterm birth in women with threatened preterm labor: A meta-analysis. Ultrasound. Obstet. Gynecol..

[5] Berghella V (2012). Universal cervical length screening for prediction and prevention of preterm birth. Obstet. Gynecol. Surv..

[6] Honest H, Forbes C, Durée K, Norman G, Duffy S, Tsourapas A (2009). Screening to prevent spontaneous preterm birth: Systematic reviews of accuracy and effectiveness literature with economic modelling. Health Technol. Assess..

[7] Leitich H, Kaider A (2003). Fetal fibronectin—How useful is it in the prediction of preterm birth?. BJOG Int. J. Obstetr. Gynaecol..

[8] Romero R, Dey SK, Fisher SJ (2014). Preterm labor: One syndrome, many causes. Science.

[9] Romero R, Hassan SS, Gajer P, Tarca AL, Fadrosh DW, Nikita L (2014). The composition and stability of the vaginal microbiota of normal pregnant women is different from that of non-pregnant women. Microbiome.

[10] Fettweis JM, Serrano MG, Brooks JP, Edwards DJ, Girerd PH, Parikh HI (2019). The vaginal microbiome and preterm birth. Nat. Med..

[11] Han YW, Wang X (2013). Mobile microbiome: Oral bacteria in extra-oral infections and inflammation. J. Dent. Res..

[12] Kindinger LM, Bennett PR, Lee YS, Marchesi JR, Smith A, Cacciatore S (2017). The interaction between vaginal microbiota, cervical length, and vaginal progesterone treatment for preterm birth risk. Microbiome.

[13] Offenbacher S, Katz V, Fertik G, Collins J, Boyd D, Maynor G (1996). Periodontal infection as a possible risk factor for preterm low birth weight. J. Periodontol..

[14] Bolyen E, Rideout JR, Dillon MR, Bokulich NA, Abnet CC, Al-Ghalith GA (2019). Reproducible, interactive, scalable and extensible microbiome data science using QIIME 2. Nat. Biotechnol..

[15] Callahan BJ, McMurdie PJ, Rosen MJ, Han AW, Johnson AJ, Holmes SP (2016). DADA2: High-resolution sample inference from Illumina amplicon data. Nat. Methods.

[16] Chen T, Yu WH, Izard J, Baranova OV, Lakshmanan A, Dewhirst FE (2010). The human oral microbiome database: A web accessible resource for investigating oral microbe taxonomic and genomic information. Database (Oxford).

[17] Love MI, Huber W, Anders S (2014). Moderated estimation of fold change and dispersion for RNA-seq data with DESeq2. Genome Biol..

[18] Breiman L (2001). Random forests. Mach. Learn..

[19] Ide M, Papapanou PN (2013). Epidemiology of association between maternal periodontal disease and adverse pregnancy outcomes–systematic review. J. Clin. Periodontol..

[20] Vander Haar EL, So J, Gyamfi-Bannerman C, Han YW (2018). Fusobacterium nucleatum and adverse pregnancy outcomes: Epidemiological and mechanistic evidence. Anaerobe.

[21] Witkin SS (2019). Vaginal microbiome studies in pregnancy must also analyse host factors. BJOG..

[22] Doyle RM, Alber DG, Jones HE, Harris K, Fitzgerald F, Peebles D (2014). Term and preterm labour are associated with distinct microbial community structures in placental membranes which are independent of mode of delivery. Placenta.

[23] Leon R, Silva N, Ovalle A, Chaparro A, Ahumada A, Gajardo M (2007). Detection of Porphyromonas gingivalis in the amniotic fluid in pregnant women with a diagnosis of threatened premature labor. J. Periodontol..

[24] Katz J, Chegini N, Shiverick KT, Lamont RJ (2009). Localization of *P. gingivalis* in preterm delivery placenta. J. Dent. Res..

[25] Yang I, Claussen H, Arthur RA, Hertzberg VS, Geurs N, Corwin EJ (2022). Subgingival microbiome in pregnancy and a potential relationship to early term birth. Front. Cell Infect. Microbiol..

[26] Basavaprabhu HN, Sonu KS, Prabha R (2020). Mechanistic insights into the action of probiotics against bacterial vaginosis and its mediated preterm birth: An overview. Microb. Pathog..

[27] Payne MS, Newnham JP, Doherty DA, Furfaro LL, Pendal NL, Loh DE (2021). A specific bacterial DNA signature in the vagina of Australian women in midpregnancy predicts high risk of spontaneous preterm birth (the Predict1000 study). Am. J. Obstet. Gynecol..

[28] Hajishengallis G (2015). Periodontitis: From microbial immune subversion to systemic inflammation. Nat. Rev. Immunol..

[29] Stout MJ, Conlon B, Landeau M, Lee I, Bower C, Zhao Q (2013). Identification of intracellular bacteria in the basal plate of the human placenta in term and preterm gestations. Am. J. Obstet. Gynecol..

[30] Aagaard K, Ma J, Antony KM, Ganu R, PetroSsino J, Versalovic J (2014). The placenta harbors a unique microbiome. Sci. Transl. Med..

