# Supplementary figures and images for: Predicting preterm birth using machine learning techniques in oral microbiome
Source: Sci Rep. 2023 Nov 30;13:21105. doi: 10.1038/s41598-023-48466-x (PMC10689490; doi:10.1038/s41598-023-48466-x)

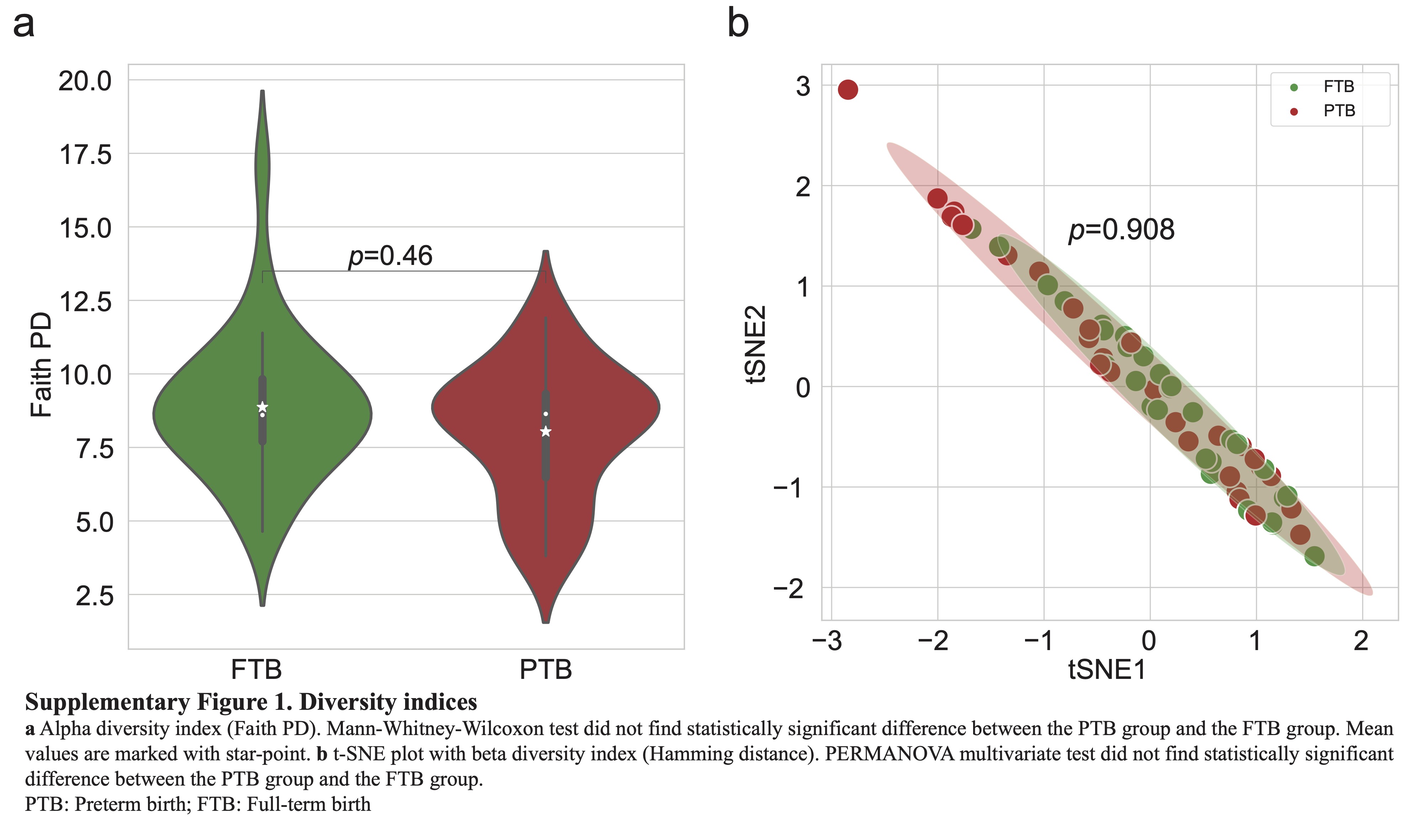

Supplement: Supplementary file 1 — Supplementary Figure 1. [file 41598_2023_48466_MOESM1_ESM.jpg]

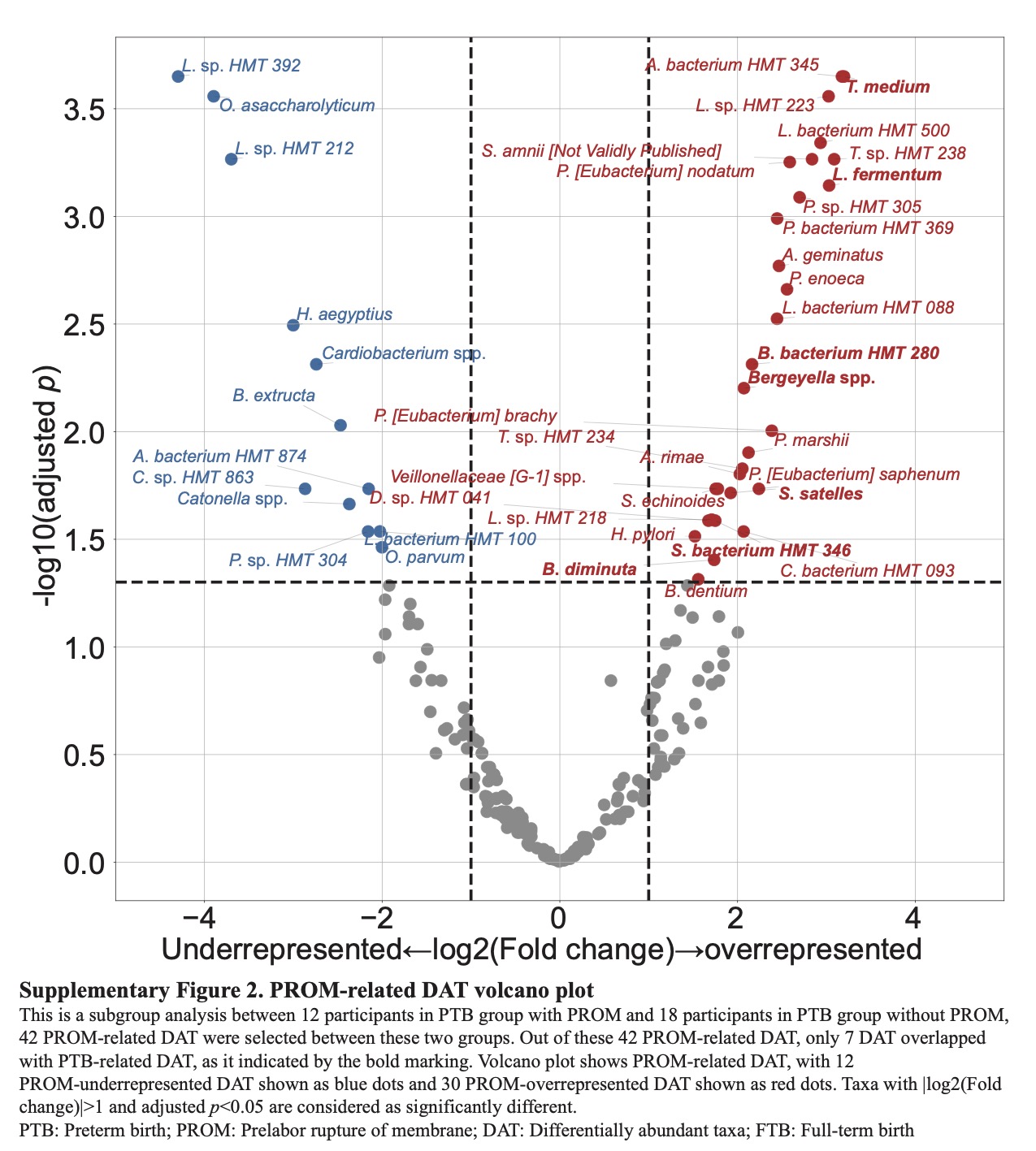

Supplement: Supplementary file 2 — Supplementary Figure 2. [file 41598_2023_48466_MOESM2_ESM.jpg]

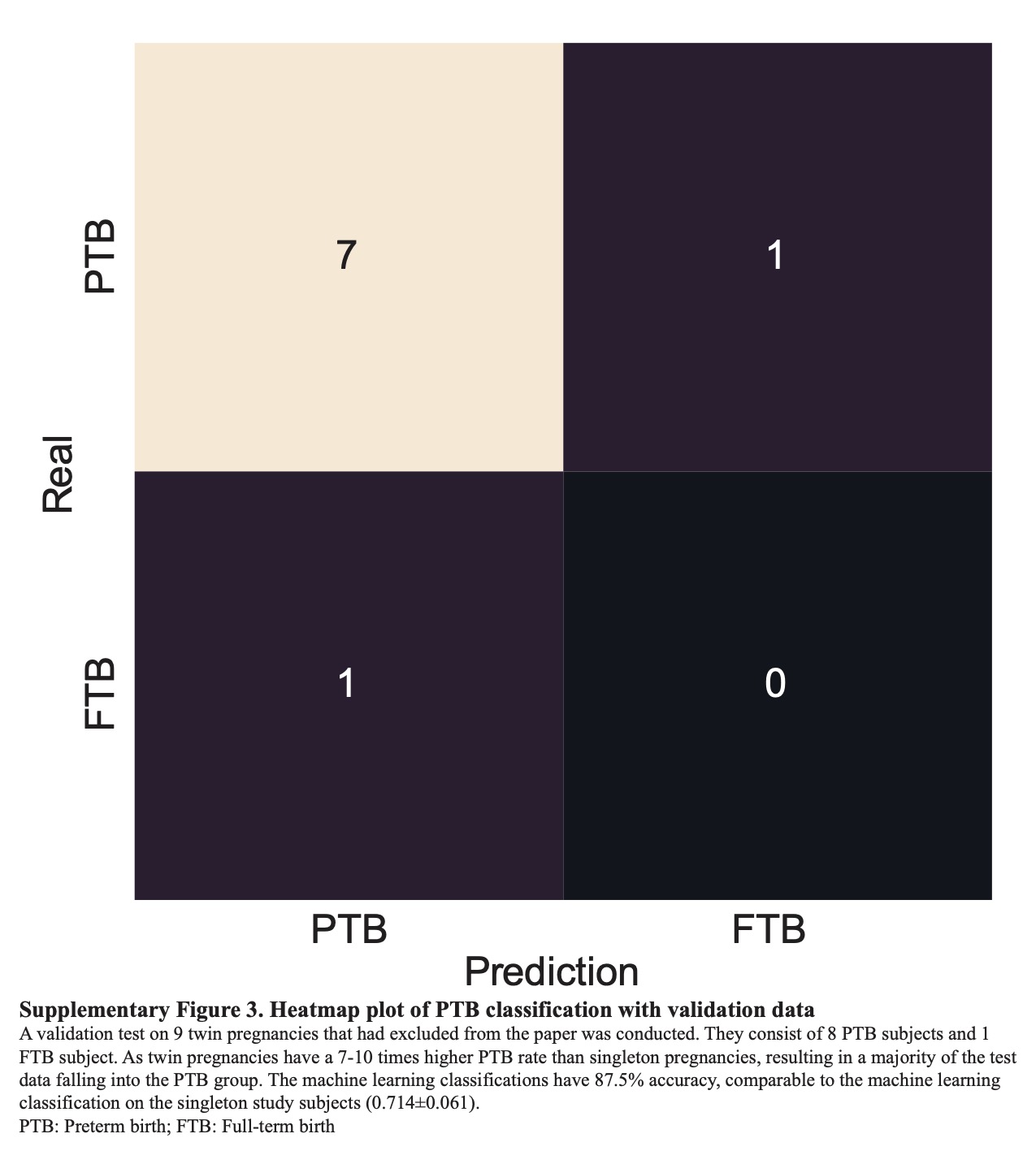

Supplement: Supplementary file 3 — Supplementary Figure 3. [file 41598_2023_48466_MOESM3_ESM.jpg]
